# Twenty-Year Summary of Surveillance for Human Hantavirus Infections, United States

**DOI:** 10.3201/eid1912.131217

**Published:** 2013-12

**Authors:** Barbara Knust, Pierre E. Rollin

**Affiliations:** Centers for Disease Control and Prevention, Atlanta, Georgia, USA

**Keywords:** hantavirus, viruses, zoonoses, Sin Nombre virus, Seoul virus, Monongahela virus, New York virus, Bayou virus, Black Creek Canal virus, hantavirus pulmonary syndrome, hemorrhagic fever with renal syndrome, respiratory infections, United States

## Abstract

In the past 20 years of surveillance for hantavirus in humans in the United States, 624 cases of hantavirus pulmonary syndrome (HPS) have been reported, 96% of which occurred in states west of the Mississippi River. Most hantavirus infections are caused by Sin Nombre virus, but cases of HPS caused by Bayou, Black Creek Canal, Monongahela, and New York viruses have been reported, and cases of domestically acquired hemorrhagic fever and renal syndrome caused by Seoul virus have also occurred. Rarely, hantavirus infections result in mild illness that does not progress to HPS. Continued testing and surveillance of clinical cases in humans will improve our understanding of the etiologic agents involved and the spectrum of diseases.

In 1993, an outbreak of severe respiratory illness in the Four Corners region of the United States (defined by the shared borders between the states of New Mexico, Arizona, Colorado, and Utah) made national headlines. The subsequent discovery of a new disease, hantavirus pulmonary syndrome (HPS) (*1*), its etiologic agent, Sin Nombre virus (SNV) (*2*), and its rodent reservoir, the deer mouse (*Peromyscus maniculatus*) (*3*), were among the most prominent findings in a flood of new revelations about hantaviruses in the Americas. Reliable and rapid diagnostic tests coupled with national surveillance created conditions whereby patients were tested, relevant information was gathered regarding rodent exposures, and potential rodent virus hosts were caught and tested. Within a few years, 4 additional disease-associated hantaviruses indigenous to the United States were described: Bayou, Black Creek Canal, New York, and Monongahela viruses. We now understand that several hantaviruses are endemic to North America, and infections in humans continue to occur where humans come into contact with infected rodents. Through a review of literature and data from the hantavirus surveillance registry of the Centers for Disease Control and Prevention (CDC), Atlanta, Georgia, USA, we have summarized the spectrum and distribution of human hantavirus infections in the United States through July 9, 2013.

Hantaviruses (family *Bunyaviridae*, genus *Hantavirus*) are rodent- and insectivore-borne viruses that are distributed in all continents except Antarctica (*4*). It is not known if all hantaviruses cause disease in humans. Humans become infected through direct contact with infected rodents or inhalation of virus that has been shed in rodent excreta, blood, and saliva and then become aerosolized. Old World hantaviruses that are known human pathogens (e.g., Hantaan, Dobrava, Seoul, and Puumala viruses) cause hemorrhagic fever and renal syndrome (HFRS), in which the primary organ affected is the kidney. Symptoms include fever; myalgia; and gastrointestinal, urinary, cerebral, and conjunctival hemorrhage. Acute renal failure with oliguria caused by HFRS often lasts for several days before spontaneously resolving (*5*). All known New World hantaviruses pathogenic to humans, including SNV and Andes virus, cause HPS, in which the primary organ affected is the lungs. Symptoms include a prodrome of fever, myalgia, and gastrointestinal symptoms followed by a rapid onset of pulmonary edema (*1*,*6*,*7*). Because cardiac insufficiency, leading to cardiac failure and death, can be prominent in severe cases, many investigators and clinicians refer to the disease as hantavirus cardiopulmonary syndrome (*8*,*9*). Thrombocytopenia, left shift, and hemoconcentration are typical abnormal laboratory findings (*10*). The only hantavirus species with evidence of human-to-human transmission is Andes virus, which is endemic to South America (*5*). In the United States, ≈90% of hantavirus infections are acquired through household or occupational exposures (*11*), although a 2012 outbreak among Yosemite National Park visitors is a noteworthy example of recreational hantavirus exposure (*12*).

National surveillance for hantavirus infections in the United States began in 1993, and HPS became nationally notifiable in 1995 (*13*). The clinical case definition, as approved by the Council of State and Territorial Epidemiologists, includes fever and pulmonary symptoms (bilateral diffuse interstitial edema, clinical diagnosis of acute respiratory distress syndrome, or radiographic evidence of noncardiogenic pulmonary edema) or unexplained respiratory illness resulting in death and an autopsy examination demonstrating noncardiogenic pulmonary edema without identifiable cause (*14*). Clinically compatible HPS cases are confirmed by laboratory testing (serologic analysis, PCR, or immunohistochemical analysis) with results positive for hantavirus infection. Laboratory confirmation is required for a case to be reported through the Nationally Notifiable Diseases Surveillance System.

As of July 9, 2013, there have been 624 reported cases of HPS in 34 states (Figure), including 31 cases that occurred before 1993 and were retrospectively diagnosed from archived autopsy tissues or convalescent serum samples (*15*–*17*). Exposure location was determined for 593 cases, and 570 (96%) of these exposures occurred in states west of the Mississippi River. Twelve infections were confirmed by PCR analysis to be caused by hantavirus species other than SNV (Table): 5 infections were caused by Bayou virus, 1 by Black Creek Canal virus, 2 by New York virus, and 4 by Monongahela virus. Because available serologic tests are broadly cross-reactive for all New World hantaviruses (*18*,*19*), virus identification must be performed by PCR with sequencing; this method requires acute specimens to be collected and transported to the laboratory frozen to preserve RNA for analysis. This is not always possible, but attempts to collect specimens suitable for molecular analysis can greatly contribute to our further understanding of the hantaviruses causing disease in the United States.

**Figure:**
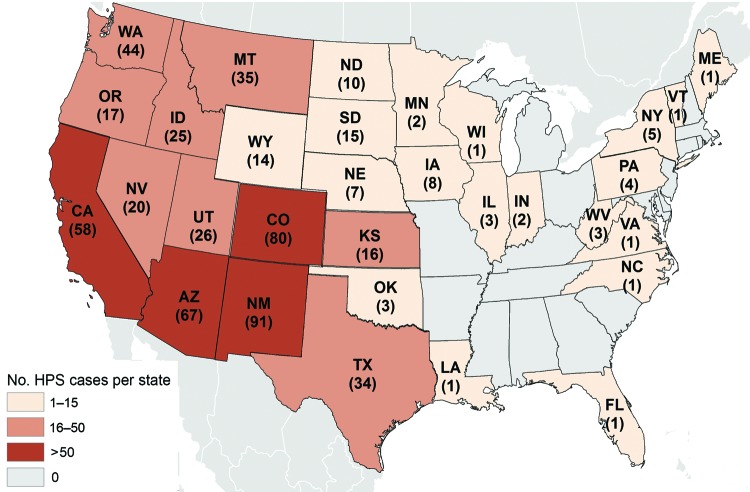
Hantavirus pulmonary syndrome (HPS) cases by state of exposure, United States, 1993–2013. A total of 624 cases occurred in 34 states; the state of exposure was unknown for another 28 cases. The cumulative case count for each state was current as of July 9, 2013.

**Table:** Summary of laboratory-confirmed hantavirus pulmonary syndrome cases caused by viruses other than Sin Nombre virus, United States, 1993–2007

Virus, reservoir, state	No. cases	Year	References
Bayou, Marsh rice rat (*Oryzomys palustris*)			(*18**,**20**,**21*)
Texas	4	1995, 1996, 2001, 2007	
Louisiana	1	1993	
Black Creek Canal, Hispid cotton rat (*Sigmodon hispidus*)			(*19*)
Florida	1	1993	
New York, White-footed mouse (*Peromyscus leucopus*)			(*22**,**23*)
New York	2	1994, 1995	
Monongahela, Deer mouse (*P. maniculatus*)			(*24**–**26*)
Pennsylvania	2	1997	
West Virginia	2	2004	

CDC has recorded a total of 10 laboratory confirmed cases of acute hantavirus infection that did not fit the clinical case definition of HPS, 6 of which clinical case descriptions have been reported previously (*12*,*27*,*28*). Because these patients did not have pulmonary symptoms, they were not included in the national HPS case counts. It is believed that human hantavirus infections infrequently result in mild illness without pulmonary symptoms, an assertion that was strengthened in the wake of the 2012 Yosemite outbreak, where only 2 acute laboratory-confirmed hantavirus infections without pulmonary symptoms were identified, in spite of extensive serologic testing—more than 3,000 samples were tested at 1 commercial laboratory alone (*29*). However, as long as pulmonary symptoms are a required element for reporting of hantavirus cases to CDC (and likely also to state public health authorities), these milder cases of hantavirus infection will continue to go uncounted. This presents a missed opportunity in understanding the full spectrum of hantavirus disease, and reduced awareness of where and how persons are exposed to hantaviruses. In this light, we propose that the clinical case definition should be adjusted so that all laboratory-confirmed hantavirus infections are reportable to health authorities.

Old World hantaviruses have also been found in this country. For example, Seoul virus has been found in Norway rats (*Rattus nor*v*egicus*) and black rats (*Rattus rattus*) in urban areas throughout the United States (*30*,*31*). A series of serosurveys in Baltimore, Maryland, residents demonstrated that humans had serologic evidence of exposure to Seoul virus and identified 3 cases that fit the clinical criteria of acute HFRS (*32*). In 2008, the first domestically acquired Seoul virus infection to be confirmed by PCR was discovered in a Baltimore resident (*33*). An unconfirmed HPS case attributed to domestically acquired Seoul virus infection in Texas in 2010 has also been described (*34*). HFRS should be considered in cases of fever and acute renal failure in persons living or working in environments that may have rats.

Imported cases of hantavirus infection have also occurred in the United States; the first were described in the 1950s when HFRS was detected in 2 military personnel returning from the Korean War (*35*,*36*). More recently, in 2009, HFRS resulting from an imported case of Seoul virus infection developed in a Wisconsin resident shortly after he returned from a visit to China (*37*); in 2010, serologically confirmed HPS resulting from an infection acquired in Brazil developed in a Brazilian visitor to Florida (*38*); and in 2012, HFRS resulting from Puumala virus infection acquired in Germany developed in a German visitor to Florida.

HPS caused by SNV continues to be the predominant form of hantavirus infection in the United States. However, we must continue to consider hantaviruses as a cause of disease in patients with rodent exposures that are outside the western United States, that differ from the usual clinical presentation of pulmonary disease, or that are not associated with deer mouse exposure.
